# Editorial: Telemedicine and telementoring in urology practice

**DOI:** 10.3389/fsurg.2022.1115671

**Published:** 2023-01-09

**Authors:** Gabriele Tulone, Nicola Pavan, M. Carmen Mir

**Affiliations:** ^1^ Department of Surgical, Oncological and Oral Sciences, Section of Urology, University of Palermo, Palermo, Italy; ^2^ Department of Urology, IMED Hospitals, Valencia, Spain

**Keywords:** telemedicine (keywords), urology, telementoring, surgery, COVID-19

**Editorial on the Research Topic** Telemedicine and telementoring in urology practice

In recent years there has been a notable increase in the spread of telemedicine as a tool for providing health care to populations with limited care access. The first applications of this practice date back to 1970 with an increasing expansion with greater portability, improved usability, lower costs, and higher quality thanks also to technological improvements (1). Telemedicine is defined as “medical care at a distance”. It allows real time communication between the patient and the physician. The spread of the SARS COV 2 virus has boosted this practice in order to guarantee continuity of care and access for patients in isolation. The benefits are the reduction of coronavirus spread and contamination of uninfected person. It facilitates health care professionals who are positive for COVID-19 or at high risk for adverse outcomes to continue to work while they are still isolated at home. It simplifies clinic visits for patients and, probably on the long run, can increase efficiency as part of the armamentarium for clinicians. Recent patient satisfaction evaluations show that patients are embracing telemedicine to a surprising degree, particularly for follow-up care (2, 3). Furthermore these new practice have important economic implication to reduce health care costs (1). Telementoring and telesurgery are examples of such new technologies, and they are ideally suited for use in the technology-heavy specialty that is modern urology (4). The term “telementoring” is used to describe the guidance of one health-care professional (in this case a surgeon) by another in a different location during a procedure or clinical episode. Equivalence to in-person care and high levels of patient and caregiver satisfaction are found in most studies (5, 6). Often the word “telemedicine” is used interchangeably with “telehealth” but the former refers specifically to applications used in the diagnosis and treatment of diseases, the latter is defined as a tool for remote clinical health care, vocational education and public health (7). The applications of telemedicine can take place synchronously, asynchronously or combined with in-person care. Patient and physician can interact virtually *via* fully interactive video technology in real time or asynchronously by storing and forwarding clinical data items, such as medical reports, images and video recordings, for later interpretation. In addition to clinical services, telehealth can refer to remote non-clinical services such as provider training, administrative meetings, and continuing medical education. Telemedicine has been adopted in various specialties including urology. It finds application both in oncology and in benign pathologies like renal cell carcinoma, BPH, sexual health, renal stones, urinary tract infection with proven efficiency and safety (8). A 2020 cross-sectional analysis found that the use of telemedicine in clinical urology practices across the globe has nearly tripled during the pandemic (9). More studies are needed on other highly prevalent urological malignant and benign conditions.

This Research Topic therefore provides a great opportunity to highlight and promote research in this area. It is a precise and careful collection of clinical studies and reviews on the use of telemedicine in the urological field.

First; an original research by Ziyu Liu describes the developed and tested EPSS, an innovative electronic audiovisual version of VPSS. It demonstrated to be well comprehended and accepted by patients with LUTS, and it also showed a significant correlation with the VPSS and assessment by urology specialists. The EPSS represents a valid option for the assessment of male LUTS and may be particularly indicated for telemedicine services.

Next, a systematic review by Christian Habib Ayoub provide an up-to-date about telemedicine focusing specifically on aspects related to telementoring, telestration, and telesurgery. Furthermore; in this manuscript the authors discuss the historical role in healthcare with a special emphasis on current and future use in urology.

Another review by Nithesh Naik et al. identifies that telemedicine in urology holds promise as a powerful medium for the delivery of uninterrupted high-quality urological care to patients.

Overall, thanks to the drafting of these articles, it is possible to have a clearer picture of the spread of telemedicine and telementoring in the urological field and of their reliability and safety to ensure high quality in-person care; especially in patients with difficulty in moving or in isolation. Despite the advantages associated with telemedicine, a number of significant technical and ethical issues are raised by the continued development of these practices and technologies.
